# Effective Viscous Damping Enables Morphological Computation in Legged Locomotion

**DOI:** 10.3389/frobt.2020.00110

**Published:** 2020-08-28

**Authors:** An Mo, Fabio Izzi, Daniel F. B. Haeufle, Alexander Badri-Spröwitz

**Affiliations:** ^1^Dynamic Locomotion Group, Max Planck Institute for Intelligent Systems, Stuttgart, Germany; ^2^Multi-Level Modeling in Motor Control and Rehabilitation Robotics, Hertie-Institute for Clinical Brain Research, University of Tübingen, Tübingen, Germany; ^3^Centre for Integrative Neuroscience, University of Tübingen, Tübingen, Germany

**Keywords:** damping, energy dissipation, legged locomotion, ground disturbance, drop test, rolling diaphragm

## Abstract

Muscle models and animal observations suggest that physical damping is beneficial for stabilization. Still, only a few implementations of physical damping exist in compliant robotic legged locomotion. It remains unclear how physical damping can be exploited for locomotion tasks, while its advantages as sensor-free, adaptive force- and negative work-producing actuators are promising. In a simplified numerical leg model, we studied the energy dissipation from viscous and Coulomb damping during vertical drops with ground-level perturbations. A parallel spring- damper is engaged between touch-down and mid-stance, and its damper auto-decouples from mid-stance to takeoff. Our simulations indicate that an adjustable and viscous damper is desired. In hardware we explored effective viscous damping and adjustability, and quantified the dissipated energy. We tested two mechanical, leg-mounted damping mechanisms: a commercial hydraulic damper, and a custom-made pneumatic damper. The pneumatic damper exploits a rolling diaphragm with an adjustable orifice, minimizing Coulomb damping effects while permitting adjustable resistance. Experimental results show that the leg-mounted, hydraulic damper exhibits the most effective viscous damping. Adjusting the orifice setting did not result in substantial changes of dissipated energy per drop, unlike adjusting the damping parameters in the numerical model. Consequently, we also emphasize the importance of characterizing physical dampers during real legged impacts to evaluate their effectiveness for compliant legged locomotion.

## Introduction

**Figure 1:**
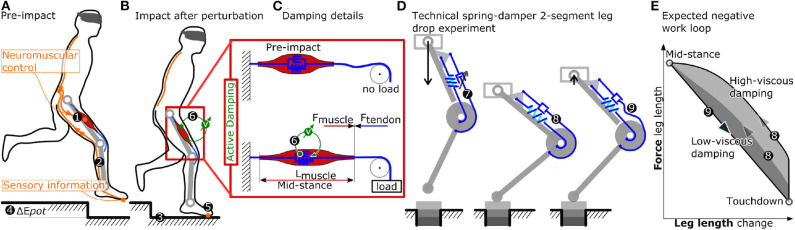
**(A–C)** Problem identification, and related research question. The limited nerve conduction velocity in organic tissue (More et al., 2010) ❷ presents a significant hazard in legged locomotion. Local neuromuscular strategies ❻ provide an alternative means of timely and tunable force and power production. Actuators like the indicated knee extensor muscle keep the leg extended during stance phase (muscle length L_muscle_) by producing the appropriate amount of muscle force (F_muscle_), correctly timed. Neuromuscular control ❶ plays a major role in initiating and producing these active muscle forces, but works best only during unperturbed locomotion. Sensor information from foot contact travels via nerves bundles ❷ to the spinal cord, but with significant time delays in the range of *t* = 40 ms (More and Donelan, 2018, for 1 m leg length) and more. Hence, the locomotion control system can become “sensor blind” due to conduction delays, for half a stance phase, and can miss unexpected perturbations like the depicted step-down. During step-down perturbations ❸ additional energy ❹ is inserted into the system. Viscous damper-like mechanisms produce velocity dependent counter-forces, and can dissipate kinetic energy. Local neuromuscular strategies ❻ producing tunable, viscous damping forces would act instantaneously and adaptively. Such strategies ❻ could also be robust to uncontrolled and harsh impacts of the foot after perturbations ❺, better than sensor-based strategies. In this work **(D)**, we are testing and characterizing spring-damper configurations mounted to a two-segment leg structure, during rapid- and slow-drop experiments, for their feasibility to physically and instantaneously produce tunable, speed-dependent forces extending the leg. Work loops **(E)** will indicate how much effective negative work is dissipated, between touch-down and mid-stance. Prior to impact ❼ and during the leg loading ❽ the spring-damper's tendons act equally. Starting at mid-stance, the main spring extends the knee, leading to leg extension and leaving the damper's tendon slack ❾.

While less understood, damping likely plays an essential role in animal legged locomotion. Intrinsic damping forces can potentially increase the effective force output during unexpected impacts (Müller et al., 2014), reduce control effort (Haeufle et al., 2014), stabilize movements (Shen and Seipel, 2012; Secer and Saranli, 2013; Abraham et al., 2015), and reject unexpected perturbations (Haeufle et al., 2010; Kalveram et al., 2012), e.g., sudden variations in the ground level (Figure 1). Stiffness, in comparison, has been studied extensively in legged locomotion. Its benefits have been shown both in numerical simulations, e.g., through spring-loaded inverted pendulum (SLIP) models (Mochon and McMahon, 1980; Blickhan et al., 2007), and physical springy leg implementations (Spröwitz et al., 2013; Hutter et al., 2016; Ruppert and Badri-Spröwitz, 2019).

What combines both mechanical stiffness and intrinsic, mechanical damping is their sensor- and computational-free action. A spring-loaded leg joint starts building up forces exactly at the moment of impact. Mechanical stiffness, or damping, acts instantaneously, and are not subject to delays from post-processing sensor data (Grimminger et al., 2020), delays from limited nerve conductive velocities (More and Donelan, 2018), or uncertainties in the estimation of the exact timing of swing-to-stance switching (Bledt et al., 2018).

Legged robots commonly exploit *virtual damping*: actively produced and sensory-controlled negative work in the actuators (Hutter et al., 2012; Havoutis et al., 2013; Seok et al., 2015; Kalouche, 2017; Grimminger et al., 2020). Virtual damping requires high-frequency force control, and actuators mechanically and electrically capable of absorbing peaks in negative work. In comparison, mechanical damping based systems (Garcia et al., 2011; Hu et al., 2019) act instantaneously, share impact loads with the actuator when in parallel configuration, and require no sensors or control feedback. The instantaneous mechanical response of a damper is especially relevant in biological systems, where the neuronal delay may be as large as 5 % to 40 % of the duration of a stance phase (More et al., 2010). In such a short time-window, physical damping could help to reject the perturbation (Haeufle et al., 2010; Kalveram et al., 2012) by morphological computation, as it mechanically contributes to the rejection of the perturbation, a contribution that otherwise would need to be achieved by a (fast) controller (Zahedi and Ay, 2013; Ghazi-Zahedi et al., 2016). Hence, physical damping has the potential to contribute to the *morphological computation* (Zahedi and Ay, 2013; Ghazi-Zahedi et al., 2016) of a legged system.

Compared to virtual damping with proprioceptive sensing strategies (Grimminger et al., 2020), a legged robot with physical damping requires additional mechanical components, e.g., a fluidic cylinder, and the mechanics to convert linear motion to rotary output. In a cyclic locomotion task, the energy removed by any damper must also be replenished. Hence, from a naive energetic perspective, both virtual and physical damping systems are costly.

Energy dissipation in the form of negative work has been quantified in running birds, and identified as a potential strategy to “. reduce the likelihood of a catastrophic fall.” Daley and Biewener (2006, p. 185). In virtual point-based control strategies for bipedal running, positive work is inserted into hip joints, and negative work is then dissipated in equal amounts in the spring-damper leg (Drama and Spröwitz, 2020). In sum, either physical damping or virtual damping allows removing energy from a legged locomotion system. In this work, we focus on physical damping produced by a viscous damper. We aim toward an understanding of how physical damping can be exploited in legged locomotion and which requirements a damper must fulfill.

We consider two damping principles: viscous damping and Coulomb damping. Viscous damping reacts to a system motion with a force that is linearly (or non-linearly) proportional to its relative acting speed. Coulomb damping generates a constant force, largely independent from its speed (Serafin, 2004). From a control perspective, viscous damping can be beneficial for the negotiation of perturbations in locomotion as it approximates the characteristics of a differential, velocity-dependent term. Yet it is unknown how this intuition transfers into reality, where impact dynamics and non-linearities of the leg geometry alter the stance-phase dynamics of locomotion.

Damping in legged locomotion can have other purposes, besides dissipating energy. The authors of Werner et al. (2017, p. 7) introduced a damping matrix in the control scheme, which reduced unwanted oscillations in the presence of modeling errors. Tsagarakis et al. (2013) mount compliant elements with some damping characteristics, which also could reduce oscillations of the system's springy components.

**Figure 2:**
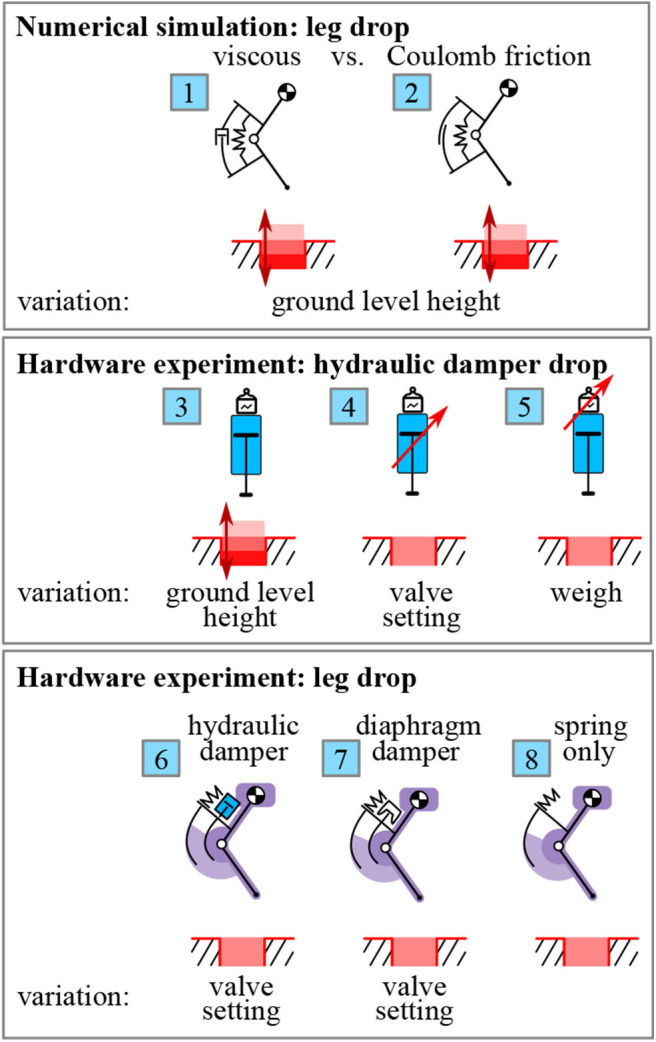
Overview: We study the *effective dissipated energy* *E*_effective_ in drop experiments, i.e., the energy dissipation within one drop cycle between touch-down and lift-off (Figure 6). We focus on a system design with a damper and a spring, both acting in parallel on the knee joint (Figures 1D, 3). No active motor is considered as it is not relevant for the drop scenario, but required for continuous hopping. In numerical simulations, we quantify the difference in energy dissipation between viscous 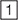 and Coulomb 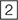 damping for varying ground level heights (section 2 and Figure 4). The first set of hardware experiments characterizes the industrial hydraulic damper. For this, we drop the isolated damper (damper only, not mounted in the leg) on a force sensor and calculate the energy dissipation. We vary the ground level height 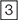, the valve setting 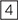 and the drop mass 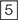, to investigate its dynamic characteristics (section 4.1 and Figure 7). For the second set of hardware experiments, we drop a 2-segment leg with dampers mounted in parallel to knee springs. We investigate the energy dissipation dynamics of the hydraulic 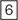 and diaphragm damper 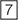 by comparing it to a spring-only condition 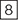, where the damper cable is simply detached (section 4.2 and Figure 8). We also vary the valve setting on the dampers to test the dynamic adjustability of damping (section 4.3 and Figure 9).

In this project, we focus our investigation on the effect of damping during the touch-down (impact) and mid-stance. We chose this simpler drop-down scenario as it captures the characteristics of roughly half a locomotion cycle. A complete cycle would require an active push off phase, and the leg's swing dynamics. Hence, we study the effectiveness of physical damping on the leg's energy dissipation within one drop (touch-down to lift-off), by quantifying its effective dissipated energy *E*_effective_. We combine insights from numerical simulations and hardware experiments (Figure 2). By studying the response of two damping strategies (viscous and Coulomb damping) in numerical drop-down simulations, we investigate how physical damping can influence the dynamics of the impact phase. We then examine how these predictions relate to hardware experiments with two functionally different, physical dampers. Hence we explore and characterize the physical damper implementations in a robot leg for their effectiveness in drop-impacts.

## Numerical Simulation

**Figure 3:**
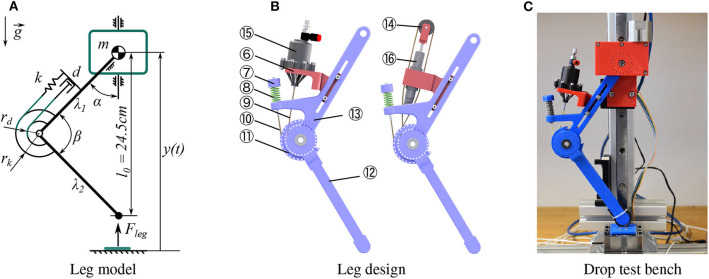
**(A)** 2-segment spring-damper-loaded leg model used for simulation. **(B)** Mechanical design of the 2-segment leg. The knee pulley ⑪ is fixed with the lower segment ⑫, coupled with the spring ⑧ and the diaphragm damper ⑮ or hydraulic damper ⑯ via cables ⑨ ⑩. **(C)** Drop test bench with the 2-segment leg.

We use numerical simulations to investigate the energy dissipation in a leg drop scenario (Figure 2). In analogy to our hardware experiment (section 3.3), a 2-segment leg with a damper and a spring in parallel on the knee joint is dropped vertically (Figure 3A). Once in contact with the ground, the knee flexes and energy is dissipated. We compare viscous vs. Coulomb damping to investigate which of these two damping strategies may be more suited for the rejection of ground-level perturbations. Also, we investigate how the adjustment of the damping characteristics influences the dissipated energy. In all the damping scenarios investigated, the system is not energy conservative. As we investigate the potential benefit of damping in the initial phase of the ground contact, i.e., from touch-down to mid-stance, we do not consider any actuation. Without actuation or control, the model's dissipated energy is not refilled, unlike in, for example, periodic hopping (Kalveram et al., 2012).

### Model

The numerical model is a modified version of the 2-segment leg proposed in Rummel and Seyfarth (2008) with an additional damper mounted in parallel to the knee-spring. The equation describing our leg dynamics is:

(1) $$\begin{array}{l} ÿ\left(t\right)=\frac{F_{leg}\left(t\right)}{m}-g \end{array}$$

where *g* is the gravitational acceleration, *m* is the leg mass (lumped at the hip), and *y* (*t*) is the time-dependent vertical position from the ground. *F*_*leg*_ (*t*) is the force transmitted to the hip mass - and the ground - through the leg structure. As such, the force depends on the current phase of the hopping cycle:

(2) $$F_{leg}\left(t\right)=\{\begin{array}{ll} 0 & \text{ , flight phase: }y\left(t\right)>l_{0}\\ \frac{y\left(t\right)}{\lambda_{1}\lambda_{2}}\frac{\tau \left(t\right)}{\sin\left(\beta \left(t\right)\right)} & \text{ , ground contact: }y\left(t\right)\leq l_{0} \end{array}$$

with segment length λ_*i*_ and knee angle β (*t*) (Figure 3A), *l*_0_ is the leg length at impact. τ (*t*) is the knee torque which is produced by the parallel spring-damper element, as in

(3) $$\begin{array}{l} \tau \left(t\right)=-kr_{k}^{2}\left(\beta \left(t\right)-\beta_{0}\right)+\tau_{d}\left(t\right) \end{array}$$

with *k* and *r*_*k*_ being the spring stiffness coefficient and lever arm, respectively. τ_*d*_ (*t*) is the damping torque, which is set to zero during leg extension, i.e., the damper is only active from impact to mid-stance:

(4) $$\begin{array}{l} \tau_{d}\left(t\right)=0\;\text{if }\dot{\beta }\left(t\right)>0 \end{array}$$

The modeled damper becomes inactive during leg extension, in accordance to our hardware: the tested physical dampers apply forces to the knee's cam via a tendon (Figure 1D, ❾), and this tendon auto-decouples during leg extension. By choosing different definitions of the damper torque τ_*d*_ (*t*), we can analyse different damper concepts. The model parameters are listed in Table 1.

**Table 1:** Simulation and hardware parameters.

**Parameters**	**Symbol**	**Value**	**Unit**
Mass	*m*	0.408	kg
Reference drop height	*h*_0_	14	cm
Spring stiffness	*k*	5900	N/m
Leg segment length	λ_1_, λ_2_	15	cm
Leg resting length	*l*_0_	24.6	cm
Knee resting angle	β_0_	110	deg
Spring lever arm	*r*_*k*_	2.5	cm
Damper lever arm	*r*_*d*_	2	cm

Simulations were performed using MATLAB (the MathWorks, Natick, MA) with ODE45 solver (absolute and relative tolerance of 10^-5^, max step size of 10^-5^ s). When searching for appropriate settings of the numerical solver, we progressively reduced error tolerances and the maximum step size until convergence of the simulation results in Table 2 to the first non-significant digit.

**Table 2:** Numerical simulation.

		***Damping coeff***.		***Step-up***	***Reference height***	***Step-down***
				***h* = *h*_0_−Δ*h* = 11.5*cm***	***h* = *h*_0_ = 14*cm***	***h* = *h*_0_ + Δ*h* = 16.5*cm***
		***d*_*v*_**	***d*_*c*_**	***E*_*D*_ (Δ*E*_*D*_/Δ*E*_*T*_)**	***E*_*D*_0__ (*E*_*D*_0__/*E*_*T*_0__)**	***E*_*D*_ (Δ*E*_*D*_/Δ*E*_*T*_)**
Set 1	Viscous	29.5 Ns/m	0 N	**82 mJ (15%)**	97 mJ (17%)	**112 mJ (15%)**
	Coulomb	0 Ns/m	7.7 N	88 mJ (9%)	97 mJ (17%)	104 mJ (7%)
Set 2	Viscous	68 Ns/m	0 N	**167 mJ (30%)**	197 mJ (35%)	**227 mJ (30%)**
	Coulomb	0 Ns/m	17.3 N	178 mJ (19%)	197 mJ (35%)	214 mJ (17%)
Set 3	Viscous	119.4 Ns/m	0 N	**249 mJ (46%)**	295 mJ (53%)	**341 mJ (46%)**
	Coulomb	0 Ns/m	29.3 N	264 mJ (31%)	295 mJ (53%)	323 mJ (28%)
Set 4	Viscous	197.1 Ns/m	0 N	**330 mJ (63%)**	393 mJ (70%)	**455 mJ (62%)**
	Coulomb	0 Ns/m	46.1 N	346 mJ (47%)	393 mJ (70%)	436 mJ (43%)
Set 5	Viscous	349.4 Ns/m	0 N	**411 mJ (81%)**	492 mJ (88%)	**572 mJ (80%)**
	Coulomb	0 Ns/m	76.3N	423 mJ (69%)	492 mJ (88%)	556 mJ (64%)

**Figure 4:**
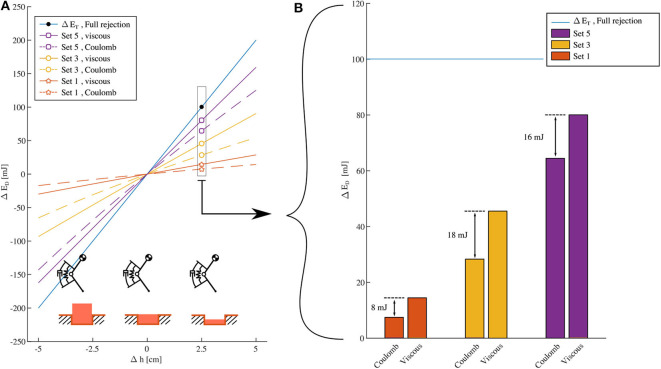
Numerical simulation Cases 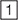 and 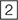 from Figure 2, **(A)**: Change of dissipated energy vs. change of drop height for set 1, 3 and 5, with damping coefficients as in Table 2. Continuous lines are viscous damping results, dashed Coulomb damping. Positive perturbations, i.e., Δ*h* > 0, correspond to step-down perturbations; step-up perturbations, otherwise. The steepest line indicates the slope needed for a *full rejection* of a Δ*h* deviation. For each set (1, 3, 5), the damping parameters are matched such that viscous and Coulomb damping dissipate the same energy at the reference height *h*_0_ (see Table 2). Within each set, the viscous damping line is closer to the desired *full rejection* line than the corresponding Coulomb damping line. This means that for the same cost (in the sense of dissipated energy at the reference height) viscous damping always rejects more of ground level perturbation than Coulomb damping. **(B)**: Δ*E*_*D*_ for Δ*h* = 2.5 cm. The horizontal line indicates the amount of energy to dissipate for *full rejection* of Δ*h*. Energetic advantage of viscous damping over Coulomb damping, as indicated by the spread in the corresponding Δ*E*_*D*_ values, increases from set 1 to 3, and reduces from set 3 to 5.

*Total dissipated energy (E_D_) in one drop cycle for different drop heights (h). Reference height is the reference drop height h = h_0_ = 14 cm. During step-up (-down) condition, the drop height is reduced(increased) by Δh = 2.5 cm. Percentage values indicate the change in dissipated energy (ΔE_D_) relative to the change in system total energy (ΔE_T_) due to the height perturbations. Each set simulates two separate mechanical dampers (pure viscous or pure Coulomb damping), with damping coefficients chosen to dissipate the same energy at the reference condition, i.e., E_D_0__. Results of set 1, 3 and 5 are further described in Figure 4. For all tested conditions, viscous damping outperforms Coulomb damping, as indicated by the always higher percentage values (bold)*.

### Damping Characteristics

We compared two damping concepts in our numerical simulation: (1) *pure* Coulomb damping, i.e., a constant resistance only dependent on motion direction, and *pure* viscous damping, i.e., a damper torque linearly dependent on the knee angular velocity. Accordingly, we tested two different definitions of τ_*d*_:

(5) $$\tau_{d}\left(t\right)=\{\begin{array}{ll} -d_{c}r_{d}\mathrm{sign}(\dot{\beta }\left(t\right)) & \;,\text{pure Coulomb damping}\\ -d_{v}r_{d}^{2}\dot{\beta }\left(t\right) & \;,\text{pure viscous damping} \end{array}$$

where *r*_*d*_ is the damper lever arm, *d*_*c*_ (in N) and *d*_*v*_ (in Ns/m) the Coulomb damping and viscous damping coefficients, respectively.

### Energy Dissipation in Numerical Drop Simulations

With this model, we investigate the difference in energy dissipation in response to step-up/down perturbations (cases 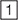 and 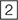 in Figure 2). For each drop test, the numerically modeled leg starts at rest (ẏ (*t*) = 0) with a drop height

(6) $$\begin{array}{l} h=y\left(t=0\right)-l_{0} \end{array}$$

corresponding to the foot clearance at release. The total energy at release is *E*_*T*_ (*h*) = *mg h*. Given that all model parameters in Table 1 are fixed, the energy dissipated in a drop becomes a function of the drop height and the damping coefficients: *E*_*D*_ = *f*_*E*_*D*__ (*h, d*_*c, v*_).

A simulated drop height *h* can be seen as a variation Δ*h* from a reference value *h*_0_:

(7) $$\begin{array}{l} h=h_{0}\pm \Delta h \end{array}$$

Equal to the hardware experiments, we use *h*_0_ = 14 cm as reference drop height. In the reference drop condition, i.e., *h* = *h*_0_, the energy dissipated by damping is *E*_*D*_0__ = *E*_*D*_ (*h*_0_) = *f*_*E*_*D*__ (*h*_0_, *d*_*c, v*_). *E*_*D*_0__ only depends on the damping level, namely the chosen damping strategy (viscous or Coulomb damping) and associated damping coefficient. We chose five different desired damping levels (set 1–5) as a means of scanning a range in which the damping could be adjusted: for each set, the amount of energy that is dissipated at the reference drop height *E*_*D*_0__ differs. The chosen *E*_*D*_0__ values (Table 2, column “Reference height”) correspond to proportional levels ([0.1, 0.2, …, 0.5]) of the systems potential energy in terms of the leg resting length *l*_0_, as in

(8) $$\begin{array}{l} E_{D_{0}}\approx mg\;[0.1,0.2,\ldots ,0.5]l_{0} \end{array}$$

This corresponds to damping configurations that dissipate between ≈ 17 % and ≈ 88 % of the system's initial potential energy at the reference height (*E*_*T*_0__ = *E*_*T*_ (*h*_0_) = *mg h*_0_ = 560 mJ), as shown in Table 2, column “Reference height.” To achieve these desired damping levels, we adjusted the damper parameters *d*_*c*_ and *d*_*v*_ accordingly (Table 2, column “Damping coeff.”). As an example: for set 3, both damping values were adjusted such that at the reference height *h*_0_ both dampers dissipate *E*_*D*_0__ = *mg* 0.3*l*_0_ = 295 mJ, which corresponds to 53 % of the total energy *E*_*T*_0__.

In the numerical simulations, we focus on the relation between a ground level perturbation Δ*h* and the change in energy dissipation – and their dependency on the damper characteristics. A drop from a height larger than *h*_0_ corresponds to a step-down (Δ*h* > 0), and a drop from a height smaller than *h*_0_ to a step-up (Δ*h* < 0). Each condition introduces a change of the total energy of Δ*E*_*T*_ = *mg Δh*. The change in energy dissipation due to the perturbation is defined as

(9) $$\begin{array}{l} \Delta E_{D}\left(\Delta h\right)=E_{D}\left(h_{0}+\Delta h\right)-E_{D_{0}} \end{array}$$

which is the difference between the dissipated energy when released from a perturbed height and the dissipated energy when released from the reference height. As a reference, we further define the *full rejection* case where

(10) $$\begin{array}{l} \Delta E_{D}\left(\Delta h\right)=\Delta E_{T}=mg\Delta h \end{array}$$

In human hopping a full recovery within a single hopping cycle is not seen during experimental drop down perturbations. Instead, a perturbation of Δ*h* = 0.1*l*_0_ is rejected in two to three hopping cycles (Kalveram et al., 2012). In our results, this corresponds to the partial rejections observed with viscous damping in sets 2 and 3 for Δ*h* = ±2.5 cm.

### Simulation Results

Figure 4A shows the relation between the change in drop height and the corresponding change in dissipated energy by the simulated dampers for set 1, 3 and 5 (continuous line for pure viscous, dashed for pure Coulomb damping). For the range of simulated drop heights, pure viscous and Coulomb dampers change the amount of dissipated energy with an almost linear dependence on the drop height. However, pure viscous damping has a slope closer to the *full rejection* scenario (blue line in Figure 4A), regardless of the set considered. In a step-down perturbation (Δ*h* > 0 in Figure 4A), pure viscous damping dissipates more of the additional energy Δ*E*_*T*_, while in a step-up perturbation (Δ*h* < 0) it dissipates less energy than pure Coulomb damping. As such, the results show that a viscous damper can reject a step-down perturbation faster, e.g., within less hopping cycles, and it requires smaller correction by active energy supply during a step-up perturbation.

Adjusting the damping parameters allows to change the reaction to a perturbation (Figure 4). Increasing the damping intensity, i.e., *d*_*v*_ and *d*_*c*_ from set 1 to 5, allows to better match the *full recovery* behavior (blue line in Figure 4A). However, this comes at the cost of a higher energy dissipation at the reference height, i.e., in absence of a ground perturbation (Table 2, column “reference height”). Increasing the damping rate also affects the energetic advantage of viscous damping over Coulomb damping. Figure 4B shows this in detail for a *specific* step-down perturbation (Δ*h* = 2.5 cm): from set 1 to set 3, the spread between the Δ*E*_*D*_ values of the viscous damper and the Coulomb damper increases (from 8 to 18 mJ). However, the difference in dissipated energy Δ*E*_*D*_ slightly reduces from set 3 to set 5 (from 18 to 16 mJ).

Table 2 quantifies the previous findings by indicating the percentage of energy perturbation Δ*E*_*T*_ that each damping approach dissipates for Δ*h* = ±2.5 cm and for all the tested sets of damping coefficients *d*_*v*_ and *d*_*c*_. The data further confirms the observations from Figure 4, showing that:

1. within each set, viscous damping outperforms Coulomb damping for all the simulated conditions - its dissipated energy is always the closest to 100 % of Δ*E*_*T*_, which means the closest to *full rejection*;
2. the energetic benefit of viscous damping over Coulomb damping, i.e., the spread in percentage values of Δ*E*_*D*_/Δ*E*_*T*_, *does not monotonically increase* with higher damping rates, i.e., moving from set 1 to 5.

Furthermore, Table 2 shows that for small damping rates, i.e., set 1, viscous damping introduces only marginal benefits in energy management compared to Coulomb damping: < 10% spread between the corresponding Δ*E*_*D*_/Δ*E*_*T*_ values.

## Hardware Description

**Figure 5:**
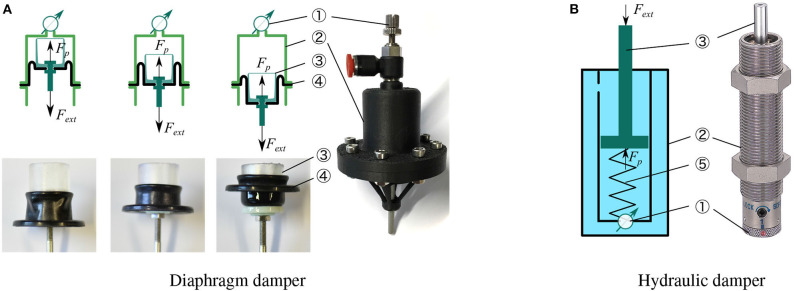
**(A)** Left-top: schematic of a diaphragm damper, illustrating the motion of rolling diaphragm, which includes an adjustable orifice ①, a cylinder ②, a piston ③, and a rolling diaphragm ④. **(B)** Right: schematic of a hydraulic damper: fluid is sealed inside the cylinder ② with an recovery spring ⑤ to reset the piston ③.

With the previous results from our numerical simulation in mind, we tested two technical implementations (Figure 5) to produce adjustable and viscous physical damping. We implemented a 2-segment leg hardware (Figure 3B) and mounted it to a vertical drop test bench to investigate the role of physical damping. The drop test bench produces velocity profiles during impact and stance phase similar to continuous hopping and allows us testing effective damping efficiently and repeatable.

### Rolling Diaphragm Damper

The most common designs of viscous dampers are based on hydraulic or pneumatic cylinders (viscous damping) and can offer the possibility of regulating fluid flow by altering the orifice opening (adjustability). These physical dampers can display high Coulomb friction, caused by the mechanical design of the sliding seal mechanisms. Typically, the higher the cylinder pressure is, the higher the Coulomb friction exists. Ideally, we wanted to test one physical damper concept with the least possible amount of Coulomb friction. Inspired by the low-friction hydrostatic actuators (Whitney et al., 2014, 2016), we designed a low-Coulomb damper based on a rolling diaphragm cylinder. Its cylinder is 3D printed from Onyx material. Figure 5A illustrates the folding movement of this rolling diaphragm mounted on a piston. The rolling diaphragm is made of an elastomer shaped like a top hat that can fold at its rim. When the piston moves out, the diaphragm envelopes the piston. In the ideal implementation, only rolling contact between the diaphragm and the cylinder occurs, and no sliding contact. Hence, Coulomb friction between piston and cylinder is minimized. We measured *F*_*C*_ ≈ 0.3 N of Coulomb friction for our rolling diaphragm cylinder, at low speed.

Our numerical simulation results promoted viscous and adjustable damping for use in vertical leg-drop. By concept, both properties are satisfied by the diaphragm damper with an adjustable valve. When an external load *F*_*ext*_ pulls the damper piston (Figure 5A), the fluid flows through a small orifice, adjustable by diameter. This flow introduces a pressure drop Δ*P*(*t*), whose magnitude depends on the orifice cross-section area *A*_*o*_ and piston speed *v* (*t*). As such, for a given cylinder cross section area *A*_*p*_, the diaphragm damper reacts to an external load *F*_*ext*_ by a viscous force *F*_*p*_(*t*) due to the pressure drop Δ*P*(*t*):

(11) $$\begin{array}{l} F_{p}\left(t\right)=A_{p}\Delta P\left(t\right)=A_{p}f\left(v\left(t\right),A_{o}\right) \end{array}$$

We mounted a manually adjustable valve (SPSNN4, MISUMI) to set the orifice size *A*_*o*_. For practical reasons (weight, leakage, complexity of a closed circuit with two cylinders) we used air in the diaphragm cylinder as the operating fluid, instead of liquid (Whitney et al., 2014, 2016). Air is compressible, and with a fully closed valve the diaphragm cylinder also acts as an air spring. This additional functionality can potentially simplify the overall leg design. With the pneumatic, rolling diaphragm-based damper implementation, we focused on creating a light-weight, adjustable damper with minimal Coulomb friction, and air as operating fluid.

### Hydraulic Damper

In the second technical implementation we applied an off-the-shelf hydraulic damper (1214H or 1210M, MISUMI, Figure 5B), i.e., a commercially available solution for adjustable and viscous damping. Tested against other hydraulic commercial dampers, we found these specific models to have the most extensive range of adjustable viscous damping and the smallest Coulomb friction (*F*_*C*_ ≈ 0.7 N). Similarly to the diaphragm damper, these hydraulic dampers produce viscous damping by the pressure drop at the adjustable orifice. The operating fluid is oil, which is in-compressible. Hence, the hydraulic damper should not exhibit compliant behavior. Other than the diaphragm damper, the hydraulic damper produces damping force when its piston is pushed, not pulled. This design also includes an internal spring to recover the piston position when unloaded. In sum, the hydraulic damper features high viscous damping, no air-spring effect, and a higher Coulomb friction compared the custom-designed pneumatic diaphragm damper.

### Articulated Leg Design

The characteristics of a viscous damper strongly depend on the speed- and force-loading profile imposed at its piston, because of the complex interaction of fluid pressure and compression, viscous friction, and cavitation (Dixon, 2008). We implemented a hardware leg to test our two physical dampers at loading profiles (speed, force) similar to legged hopping and running.

The 2-segment hardware leg (Figure 3B) is designed with a constant spring and damper lever arm, parameters are provided in Table 1. In all experiments with the 2-segmented leg, the leg spring provides elastic joint reaction forces. Dampers are swapped in and out in a modular fashion, depending on the experimental settings. The 2-segment leg design parameters are identical to those in our simulation model (Table 1). A compression spring ⑧ is mounted on the upper leg segment ⑬. When the leg flexes, the spring is charged by a spring cap ⑦ coupled to a cable ⑩ attached to the lower leg. Either damper ⑮ ⑯ is fixed on a support ⑥ on the upper segment ⑬. The support ⑥ can be moved within the upper segment ⑬, to adjust the cable ⑨ pretension. Cables ⑨ ⑩ link the damper piston ③ (Figure 5) and the spring ⑧ to the knee pulley ⑪, which is part of the lower segment ⑫.

During the leg flexion, the cable under tension transmits forces instantly to the spring and damper. Spring and damper forces counteract the knee flexion. During leg extension, the spring releases energy, while the damper is decoupled due to slackness of the cable. We included a hard stop into the knee joint to limit the maximum leg extension, and achieve a fixed leg length at impact. At maximum leg flexion at high leg loading, segments can potentially collide. We ensured not to hit either hard stops during the drop experiments. The hydraulic damper ⑯ requires a reverse mechanism ⑭, since its piston requires compression to work. The piston of the diaphragm damper ⑮ was directly connected to the knee pulley. The diaphragm damper ⑮ included no recovery spring ⑤ (Figure 5), hence we reset the piston position manually after each drop test. In sum, different spring-damper combinations can be tested with the 2-segment leg setup. Note that the here shown hardware leg has no actuation. If a motor would actuate the knee joint, in parallel mounted to the spring and the damper, the damper would share the external impact load, and consequently reduce an impact at the motor.

### Experimental Set-Up, Data Sampling, and Processing

We implemented an experimental setup for repetitive measurements (Figure 3C). A drop bench was used to constrain the leg motion to a single vertical degree of freedom, and linear motion. This allowed us to fully instrument the setup (slider position, and vertical ground reaction forces, GRF), and ensured repeatable conditions over trials. Adjusting the drop height allowed us setting the touch-down speed. A linear rail (SVR-28, MISUMI) was fixed vertically on a frame. The upper leg segment was hinged to a rail slider. The rail slider was loaded with additional, external weights, simulating different robot masses. We set the initial hip angle α_0_ to align the hip and foot vertically. A hard stop ensured that the upper leg kept a minimum angle α > α_0_.

Two sensors measured the leg dynamics: the body position *y* and the vertical ground reaction force are recorded by a linear encoder (AS5311, AMS) and a force sensor (K3D60a, ME, amplified with 9326, Burster), respectively (Figure 3C). The duration from touch-down to mid-stance is very short, typically *t* ≤ 100 ms, and high-frequency data sampling was required. The encoder data was sampled by Raspberry Pi 3B+ with f = 8 kHz sampling rate. Force data were recorded by an Arduino Uno, with a 10-bit internal ADC at 1 kHz sampling rate. A high-speed camera (Miro Lab 110, Phantom) recorded the drop sequence at f = 1 kHz sampling rate. We performed ten trials for each test condition. Sensor data was processed with MATLAB (the MathWorks, Natick, MA). Data was smoothed with a moving average filter, with a filter span of 35 samples for encoder data, and 200 samples for force data. Repeated experiments of the same test condition are summarized as an envelop defined by the average ± 95% standard deviation of the filtered signals.

## Hardware Experiments and Results

**Figure 6:**
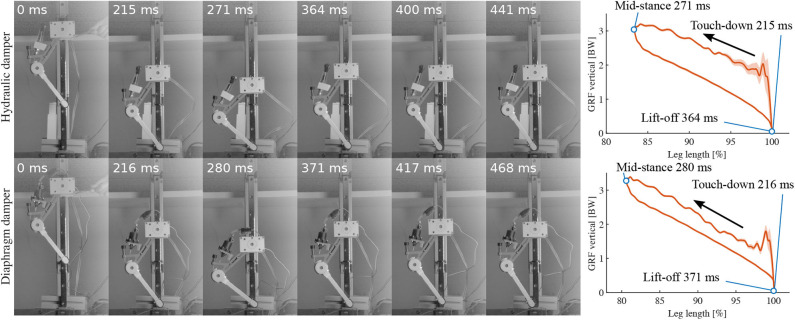
High-speed snapshots of drop experiments starting from release to second touchdown. Leg with hydraulic damper is shown on the top row, leg with diaphragm damper the bottom row. Depicted are from left to right: release, touchdown, mid-stance, lift-off, apex, second touchdown. The right plots illustrate the timing of the events corresponding to the snapshots.

In the drop experiments, we characterize both the hydraulic and diaphragm dampers, and the 2-segment springy leg (Figure 6). We chose three orifice settings (labeled as a, b, and c) for each damper, and focus on the effects of viscous damping and adjustable dissipation of energy in the hardware setup. Table 3 lists an overview of the drop tests, and its settings (drop height, weight, orifice setting, damper type). To emphasize the fundamental differences between the damper designs, we compare only one model of the hydraulic damper (1214H) to the diaphragm damper (sections 4.1.–4.3), and show the potential of the second hydraulic damper (1210M) in section 4.4. Videos of the experiments can be found in the Supplementary Material, and online^1^.

**Table 3:** Drop test settings for experiments.

**Drop test setup**	**Figure**	**Drop height**	**Drop weight**	**Orifice**
		**[cm]**	**[g]**	**[~]**
Damper (1214H)	Figure 7A	**3, 5, 7**	280	b
	Figure 7B	5	280	**a, b, c**
	Figure 7C	3	**280, 620**	b
Damper (1214H, diaphragm) & leg	Figures 8A,B	14	408	c
	Figure 8C	14	408	damper detached
Damper & leg (simulation)	Figures 9A,B	14	408	**a, c**
	Figure 9C	14	408	**viscous, Coulomb**
Damper (1210M) & leg	Figure 10	14	408	**a, b**

*Values indicated in bold indicate control parameters for these experiments*.

### Isolated Damper Drops, Evaluation

In this experiment we characterized the hydraulic damper by dropping it under changing conditions of the instrumented drop setup, without mounting it to the 2-segment leg. The experimental setup allows differentiating effects, compared to the 2-segment leg setup, and to emphasize the viscous damper behavior of the off-the-shelf component. We also applied the results to estimate the range of damping rates available with changing orifice settings. The hydraulic damper was directly fixed to the rail slider into the drop bench (section 3.4). The piston pointed downwards. We measure the vertical ground reaction force to determine the piston force, and we recorded the vertical position of the slider over time, to estimate the piston speed after it touches the force sensor.

**Figure 7:**
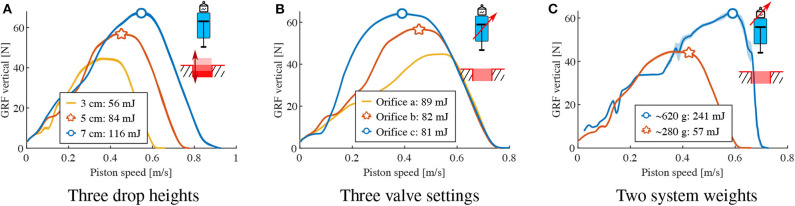
Characterizing the hydraulic damper. A single damper (not leg-mounted) drops onto the force sensor. 10 repeated experiments are plotted as an envelop, defined by the average ±95 % of the standard deviation data. The curves are read from right to left, i.e. from touch-down at maximum speed to zero speed at rest, also corresponding to the maximum damper compression. **(A)** 280 grams drop mass with medium orifice in 3 drop heights. **(B)** 280 grams drop mass with 5 cm drop height in 3 orifice settings. **(C)** Three centrimeter drop height with medium orifice in 2 drop weights.

Figure 7 shows the force-speed profiles for drop tests with different drop heights (Figure 7A), orifice settings (Figure 7B), and drop loads (Figure 7C). Data lines in Figure 7 should be interpreted from high speed (impact, right side of each plot) to low speed (end of settling phase, 0 m/s, left). The time from impact to peak force (right slope of each plot) is (≈24 ms), while the negative work (shown in legends) was mainly dissipated along the falling slope in the much longer-lasting settling phase after the peak (left slope of each plot, ≈200 ms).

The results from tests with drop heights from 3 to 7 cm show viscous damping behavior in the settling phase after peak force (left slope), with higher reaction forces at higher piston speeds with higher dissipation, ranging from 45 N for maximum speeds of 0.6 m/s with 56 mJ to 65 N at 0.9 m/s with 116 mJ. The piston force almost linearly depends on the piston speed (Figure 7A).

Changing the orifice setting at a constant drop height resulted in different settling slopes (Figure 7B). Applying a least-squares fit on the left-falling settling slope, we estimate an adjustable damping rate between 91 Ns/m and 192 Ns/m. The dissipated energy changes from 89 mJ to 81 mJ, respectively. Hence adjusting the orifice setting has an effect on the damping rate and the dissipated energy in the isolated hydraulic damper, but not as we intuitively expected.

We interpret the rising slope in the impact phase (right part of each curve, Figures 7A,B) as a build-up phase; the hydraulic damper takes time (≈24 ms) to build up its internal viscous flow and the related piston movement, after the piston impact. With heavier weights (620 g = heavy, 280 g = light, Figure 7C), the impact phase equally lasts ≈24 ms. After the impact phase with heavy weight, the damper shows the same damping rate in the settling phase, in form of an equal left slope.

Similar drop tests for the evaluation of the isolated diaphragm damper were not possible since the orientation of the internal diaphragm only permits to pull the piston. In the following section, we test the diaphragm (connected by a piston reverse mechanism) and the hydraulic damper directly on the 2-segment leg structure.

### Composition of Dissipated Energy

We performed drop tests of two damper configurations: one off-the-shelf hydraulic damper, and custom-made pneumatic damper, each mounted in parallel to a spring at the 2-segment leg (section 3.3, Figure 3B), to quantify the effect of viscous damping for drop dynamics similar to legged hopping.

For each drop, the effective dissipated energy *E*_effective_ was computed by calculating the area enclosed by the vertical GRF-leg length curve from touch-down to lift-off (Josephson, 1985), i.e., the work-loop area. These work-loops are to be read counter-clockwise, with the rising part being the loading during leg flexion, and the falling part being the unloading, due to spring recoil. *E*_effective_ does not only consist of the viscous loss *E*_viscous_ due to the damper, but also Coulomb friction loss in the leg (*E*_cfriction_) and the impact loss *E*_impact_ due to unsprung masses:

(12) $$\begin{array}{l} E_{\text{effective}}=E_{\text{cfriction}}+E_{\text{impact}}+E_{\text{viscous}}. \end{array}$$

We propose a method to indirectly calculate the contribution of viscous damping, by measuring and eliminating effects from Coulomb friction, and unsprung masses.

To quantify the Coulomb friction loss *E*_cfriction_, we conducted “slow drop” tests. The mechanical setup is identical to “free drops” test, where the leg is freely dropped from a fixed height. However, in the “slow drop” experiment the 2-segment leg is lowered manually onto the force sensor, contacting and pressing the leg-damper-spring system onto the force sensor. At slow speed only Coulomb friction in joints and damper act, but no viscous damping or impact losses occur. Consequently the dissipated energy calculated from the size of the work loop is due to Coulomb friction losses *E*_cfriction_.

**Figure 8:**
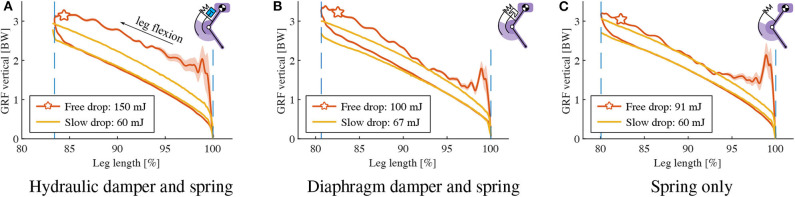
Characterizing the contribution of velocity-dependent damping: vertical GRF vs. leg length change, a 2-DOF leg with damper/spring drops onto the force sensor: Three different hardware configurations (**A**: hydraulic damper and spring, **B**: diaphragm damper and spring, **C**: spring only) were tested, for slow and free drop speeds on the vertical slider. Yellow data lines indicate slow-motion experiments. Experiments “start” bottom right, at normalized leg length 100 %. Reading goes counter-clockwise, i.e., from touch-down to mid-stance is indicated by the upper part of the hysteresis curve, while the lower part indicates elastic spring-rebound, without damper contribution.

To identify the impact loss *E*_impact_, we remove the viscous component first by detaching the damper cable on the setup. A “free drop” test in this spring only condition measures the contribution of friction loss *E*_cfriction_ and impact loss *E*_impact_ combined. A “slow drop” test of the same setup is able to quantify the friction loss *E*_cfriction_. The impact loss *E*_impact_ is therefore estimated as the energy difference between “free drop” and “slow drop” in the spring-only condition (Figure 8C). Since the effective dissipated energy *E*_effective_ is directly measured, and the friction loss *E*_cfriction_ and impact loss *E*_impact_ are obtained separately, the viscous loss *E*_viscous_ can be computed according to Equation (12).

Figures 8A,B show the “free drop” and “slow drop” results of the hydraulic damper and diaphragm damper, respectively. Both drop heights are 14 cm, at identical orifice setting. We calculated the negative work of each work-loop (range indicated by the two vertical dash lines), as shown in Figure 8. To provide an objective analysis, the work-loop area of each “slow drop” (manual movement) was cut to the maximum leg compression of the corresponding “free drop” condition. The dissipated energy of the leg-mounted hydraulic damper is 150 mJ and 60 mJ for “free drop” and “slow drop,” respectively, and 100 mJ and 67 mJ for the diaphragm damper, respectively. According to Figure 8C, the impact loss *E*_impact_ due to unsprung masses play a large role, accounting for 31 mJ. The viscous loss *E*_viscous_ of the hydraulic and the diaphragm damper are 59 mJ and 2 mJ, respectively.

### Adjustability of Dissipated Energy

**Figure 9:**
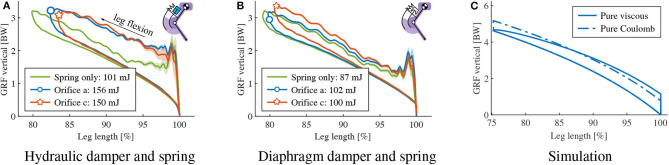
Adjustability and tunability of damping: vertical GRF vs. leg length change, a 2-segment leg with damper and spring drops onto the force sensor. Two different hardware configurations were tested, for different orifice settings. Panels **(A,B)** show the result from hydraulic damper and diaphragm damper, respectively, where the green data lines indicate the leg drop without damper for comparison. **(C)**: *Simulated* approximation of hydraulic damper orifice a by a pure viscous and a Coulomb damper. Damping coefficients are chosen to allows same dissipated energy, i.e., *E*_*D*_0__ = 156 mJ, respectively—pure viscous damper: *d*_*c*_ = 0 N and *d*_*v*_ = 51 Ns/m; pure Coulomb damper: *d*_*c*_= 13.2 N and *d*_*v*_= 0 Ns/m. None of the two curves can fully capture the work-loop of hydraulic damper.

We tested the adjustability of energy dissipation during leg drops by the altering orifice setting for each leg-mounted damper, and quantified by calculating the size of the resulting work-loops. The drop height was fixed to 14 cm and we used 2 orifice settings. The identical same set-up but in spring-only configuration (damper cables detached) was tested for reference. Work-loop and corresponding effective dissipated energies are illustrated in Figures 9A,B. The hydraulic damper-mounted leg dissipated 156 and 150 mJ energy on its two orifice settings, the pneumatic diaphragm damper dissipated 102 and 100 mJ. In Figure 9C, we display results from the numerical model introduced in section 2 to estimate the work-loop shape that either a pure viscous or pure Coulomb damper would produce, if dissipating the same amount of energy as the hydraulic damper with orifice-a (Figure 9A). We set the damping coefficients of our numerical model to *E*_*D*_0__ ≈ 156 mJ, so that: (*d*_*v*_, *d*_*c*_) = (51 Ns/m, 0 N) for pure viscous damping; and (*d*_*v*_, *d*_*c*_) = (0 Ns/m, 13.2 N) for pure Coulomb damping. Work-loops from the numerical simulation differ notably from the experimental data, suggesting that neither the hydraulic or diaphragm damper can easily be approximated as pure viscous or pure Coulomb dampers. Both work loops in Figure 9C present about equal amount of dissipated energy. Yet, both differ greatly due to their underlying damping dynamics, visible in their unique work-loop shapes. Their individual characteristics are different enough to uniquely identify pure viscous or pure Coulomb dampers, from numerical simulation.

### Damper Selection Choices

**Figure 10:**
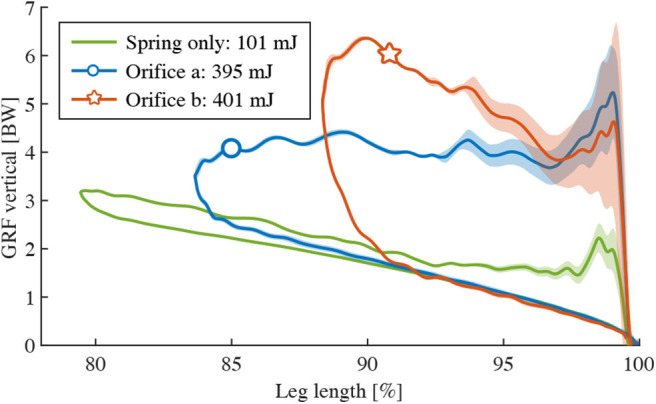
Higher energy dissipation with a different model of the hydraulic damper (1210M): Vertical GRF vs. leg length change, a 2-DOF leg with a parallel damper and spring drops onto the force sensor. Two damper orifice settings were tested (blue, red curves). The two resulting curves are compared with the spring-only configuration, provided as reference.

In accordance with the simulation results, we aim to use a viscous damper to dissipate energy introduced by a ground disturbance. How much energy could be dissipated by the damper, depended mainly on the selected viscous damper, and only to a limited degree on the orifice setting. Results from the hydraulic damper 1214H showed significant energy dissipation capabilities: ≈11 % of the system's total energy (59 of 560 mJ) were dissipated (Figure 9A at orifice setting “c” and Table 4). At the drop, in sum 150 mJ (27 %) of the leg's system energy were lost, due to Coulomb friction in the joints, impact dynamics, and viscous damping losses. Other dissipation dynamics are feasible, by selecting appropriate dampers. We tested a second hydraulic damper (1210M, MISUMI) under equal conditions and compared it to damper-1214H. The two applied orifice settings changed the observed work loop largely by shape, and little by area (Figure 10). The damper-1210M dissipated ≈60 % system energy, and the leg lost in sum (viscous+Coulomb+impact) 72 % of its system's energy during that single drop. At other orifice settings, we observed over-damping; the 1210M-spring leg came to an early and complete stop, and without rebound (data not shown here due to incomplete work loop).

**Table 4:** Leg drop experiments and their individual energetic losses per drop.

**Drop test setup**	***E*_effective_**	***E*_cfriction_**	***E*_impact_**	***E*_viscous_**
	**[mJ]**	**[mJ]**	**[mJ]**	**[mJ]**
Spring only	91	60	31	0
Diaphragm + spring	100	67	31	2
Hydraulic 1214H + spring	150	60	31	59
Hydraulic 1210M + spring	401	70	31	300

*The system's initial potential energy is 560 mJ. E_effective_, sum of all energetic losses visible as the area of the hysteresis curve; i.e., in Figure 8, E_cfriction_, negative work dissipated by Coulomb friction; E_impact_, energetic losses from impact (unsprung mass). The negative work dissipated by viscous damping in the physical damper is E_viscous_. The corresponding work curves are provided in Figures 8–10*.

**Figure 11:**
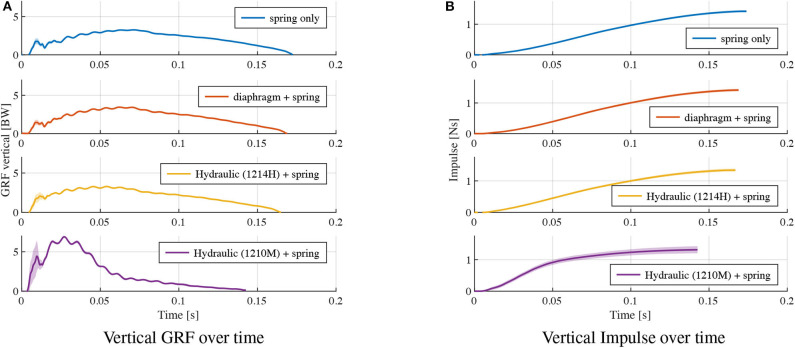
Ground reaction forces: **(A)** shows the vertical GRF and **(B)** the corresponding instantaneous vertical impulse over time, for the leg drop experiments in Figures 8–10.

For comparison, time plots of the vertical GRF and the impulse at stance phrase are shown in Figure 11. The energy composition (Equation 12) is provided in Table 4. The “spring only” data correspond the curves in Figure 8C. The diaphragm+spring data correspond to “orifice c” in Figure 9B. The hydraulic (1214H)+spring data correspond to “orifice c” in Figure 9A. The hydraulic (1210M)+spring data correspond to “orifice b” in Figure 10. Among the tested dampers, the hydraulic 1210M damper showed the largest vertical GRF; peak vertical GRF of 6.3 BW are observed, almost twice as much as the “spring only” case. The viscous dampers 1214H and 1210M shifted the peak of their legs' vertical GRF to an earlier point in time, compared to the spring-leg and the spring+diaphragm-leg (Figure 11).

## Discussion

A primary objective of this study was to test how physical dampers could be exploited for locomotion tasks by characterizing multiple available technical solutions. Our numerical model predicted three crucial aspects: (1) a pure viscous damper generally performs better than a pure Coulomb damper (Figure 4); (2) higher damping rates result in better rejection of ground disturbances (Figure 4A), however at the cost of higher dissipation at reference height (Table 2); (3) characteristic work loop shapes for pure viscous and Coulomb damper during leg-drop (Figure 9C). Our hardware findings show that neither of the tested physical dampers approximates as pure viscous or pure Coulomb dampers. The experiments also suggest that the mapping between dissipated energy and damping rates is concealed by the dynamics of the impact and the non-linearity of the force-velocity characteristics of the leg in the stance phase. Therefore, it is vital to test damping in a real leg at impact because the behavior is not merely as expected from the data sheets and the simple model.

Figure 7 characterizes how the hydraulic damper dissipates energy during a free drop. The experimental results show that the dissipated energy of the hydraulic damper scales with drop height (Figure 7A) and weight (Figure 7C), but less intuitively, it reduces with increasing damping rates (Figure 7B). This can be partially interpreted in the context of an ideal viscous damper for which the effective dissipated energy *E*_effective_ would be calculated as in,

(13) $$\begin{array}{l} E_{\text{effective}}=\int F_{p}\left(t\right)dy_{p}=\int \left(d_{v}\cdot v_{p}\left(t\right)\right)dy_{p} \end{array}$$

where *F*_*p*_(*t*) is the damper piston force and *y*_*p*_ is the piston displacement, *v*_*p*_(*t*) the corresponding velocity. When increasing the drop height, the velocity at impact is increased, so is *v*_*p*_(*t*). With the assumption of Equation (13), this results in higher damping forces *F*_*p*_(*t*), and thus, dissipated energy *E*_effective_, as seen in Figure 7A. The heavier drop weight leads to slower deceleration. Therefore the velocity profile *v*_*p*_(*t*) is increased, which also leads to higher dissipation *E*_effective_ (Figure 7B). An orifice setting of high damping rate will increase the damping coefficient *d*_*v*_. However, the velocity profile *v*_*p*_(*t*) is expected to reduce due to higher resistance. This simple analogy shows that the coupling between damping coefficient *d*_*v*_ and velocity profile *v*_*p*_(*t*) makes it difficult to predict the energy dissipation by setting the orifice and serves as an interpretation of why adjusting the orifice generates a relatively small adjustment of 10 % (81 mJ-89 mJ) of the dissipated energy. Also, the impact phase (time for the damper to output its designed damping force under sudden load) introduces additional non-linearity to the output force profile. Overall, the results in Figure 7 indicate that we can approximate the damping force produced by the hydraulic damper to be viscous and adjustable— as such dampers are typically designed (Dixon, 2008)—, but the mapping of energy dissipation to orifice setting is difficult to predict in a dynamic scenario.

The approximation as a linear, velocity dependent damper allows us to rapidly estimate energy dissipation in simulation, over a range of parameters. However, the exact mapping of the hardware leg/spring/damper energy dissipation to orifice setting is difficult to predict, when basing the estimation only on the isolated-damper drop experiments from Figure 7. Instead, the leg/spring/damper experiments show that the energetic losses from the impact remove 31 mJ energy, compared to 59 mJ damper losses. The high amount of force oscillations at impact (up to 1 BW, Figure 8A) during the first 3 % leg length change leads us to believe that these impact oscillations move the damper's dynamic working range, i.e., its resulting instantaneous force and velocity. The oscillations are likely caused by unsprung mass effects of the leg/spring/damper structure, and could not be captured in an isolated-damper setup, or—at least not easily—in a simulation.

The work loops of leg drop experiments (Figure 8) show the effects of our tested dampers on a legged system. From touch-down to mid-stance (*leg flexion*), the “free drop” curves show a larger negative work compared to the “slow drop” curves, illustrating that the damper absorbs extra energy. The returning curves (mid-stance to lift-off) of the hydraulic damper aligns well with the “slow drop” curve, indicating the damper is successfully detached due to slackness cable while the spring recoil. Figure 8B shows that the “free drop” force of the diaphragm damper is slightly higher than “slow drop” force in the first half of the leg extension phase. This discrepancy is likely caused by the elastic force component of the diaphragm damper due to sudden expansion of the air chamber volume. The elastic component seems to dominate the damper behavior, which thus acts mostly as an air spring. By separating its energetic components (Equation 12), we found that the hydraulic damper produces a viscous-like resistance higher than the diaphragm damper (59 vs. 2 mJ), indicating the hydraulic damper is more effective in dissipating energy under drop impact. Hence, the hydraulic damper shows more viscous behavior, while the diaphragm damper is more elastic.

Physical damping in the system comes at the cost of energy loss, and to maintain periodic hopping, it becomes necessary to replenish energy that is dissipated by damping (*E*_*D*_0__). Therefore, there is a trade-off to consider: simulation results show that higher damping results in faster rejection of ground perturbation at the price of more energy consumption at reference drop height (Table 2, Figure 4). An adjustable damper would partly address this problem: on level ground, the damping rate could be minimal, and on rough terrain increased. The adjustability of the two dampers is illustrated in Figures 9A,B. We discuss the adjustability from both energy dissipation and dynamic behavior perspectives.

Compared with the spring-only results, both the hydraulic and the diaphragm damper reduced the maximum leg flexion and dissipated more energy. The orifice setting changes the shape of the work loop differently for the two setups. For the hydraulic damper (Figure 9A), orifice setting-c shrinks the work loop from left edge, indicating more resistance is introduced by the damper to reduce leg flexion. For the diaphragm damper (Figure 9B), orifice setting-c not only shrinks the work loop, but also increases its slope. We interpret this as the elastic contribution of air: relatively fewer air enters through the smaller orifice, but instead acts as an in-parallel spring.

Concerning energy dissipation, changes of orifice settings led to relatively small changes in effective dissipated energy *E*_effective_: 150 to 156 mJ for the hydraulic damper, and 100 to 102 mJ for the diaphragm damper. Even for the other damper model (1210M), which dissipates high amounts of energy, changes in orifice setting change the work loop shape drastically, but not the dissipated energy (395 mJ vs. 401 mJ).

Similar to the isolated damper drop, the data (Figures 9A,B) shows that specific orifice settings introduce more resistance, but not necessarily lead to higher energy dissipation, for both hydraulic and diaphragm damper. However, in our simplified numerical leg model, an increase in viscous damping coefficients leads to a systematic increase of dissipated energy (Table 2), and a sharper tip at the left side of the work loop (Figure 9C). The discrepancy is likely due to the non-linear coupling between the damper mechanics and the leg dynamics in the hardware setup: (1) The damping force generated by the fluid dynamics in the orifice only approximates a linear viscosity (Dixon, 2008); (2) the impact loading on both the nonlinear leg structure and the damper. This makes the prediction of the energy dissipation not straight-forward based on our simplified numerical leg model, and points toward the need of a combined approach between simulation and hardware testing to fully understand physical damping in a legged system.

Viscous, velocity dependent damping alters the leg's loading characteristics, and leads to a peak force at the instance of touch-down. As a result, the vertical GRF is increased in the early stance phase, shifting and increasing the peak vertical GRF before mid-stance (Figure 11A). When designing a legged system with a viscous damper, its increasing load on the mechanical structure should be considered.

The selection of viscous dampers depends on the task. High damping can fully reject disturbances in a single cycle, but lower damping could have energetic benefits. Here we looked for a damper that would dissipate significant negative work ($\frac{E_{\text{viscous}}}{E_{{\text{T}}_{0}}}\approx 10\%-15\%$) in form of viscous damping. The air-filled diaphragm damper lead to insufficient energy losses (2 %), but the hydraulic dampers dissipated 10 % and 60% of the system's total energy (Table 4).

Drawing conclusions about animal locomotion based on the here presented leg-drop experiments is somewhat early. However, observations from Müller et al. (2014, Table 1, p. 2288) indicate that leg forces can increase at unexpected step-downs during locomotion experiments. Further, Kalveram et al. (2012) suggests in a comparison of experimental human hopping and numerical simulations that damping may be *the* driving ingredient in passive stabilization against ground-level perturbations. We are consequently excited about the here presented results of viscous dampers mounted in parallel to a leg's spring, producing adaptive forces without the need for sensing.

## Conclusion

We investigated the possibility to exploit physical damping in a simplified leg drop scenario as a template for the early stance phase of legged locomotion. Our results from a) numerical simulation promote the use of adjustable and viscous damping over Coulomb damping to deal with a ground perturbation by physical damping. As such, we b) tested two technical solutions in hardware: a commercial, off-the-shelf hydraulic damper, and a custom-made, rolling diaphragm damper. We dissected the observed dissipated energy from the hardware damper-spring leg drops, into its components, by experimental design. The resulting data allowed us to characterize dissipation from the early impact (unsprung-mass effects), viscous damping, Coulomb damping, and orifice adjustments *individually, and qualitatively*. The rolling diaphragm damper features low-Coulomb friction, but dissipates only low amounts of energy through viscous damping. The off-the-shelf, leg-mounted hydraulic damper did exhibit high viscous damping, and qualitatively showed the expected relationship between impact speed, output force and negative work. Changes in orifice setting showed only minor changes in overall energy dissipation, but can lead to large changes in leg length dynamics, depending on the chosen technical damper. Hence, switching between different viscous, hydraulic dampers is an interesting future option. Our results show how viscous, hydraulic dampers react velocity-dependent, and create an instantaneous, physically adaptive response to ground-level perturbations without sensory-input.

## Data Availability Statement

The datasets generated for this study are available on request to the corresponding author.

## Author Contributions

AM contributed to concept, hardware design, experimental setup, experimentation, data discussion and writing. FI contributed to concept, simulation framework, experimental setup, data discussion and writing. DH and AB-S contributed to concept, data discussion and writing.

## Conflict of Interest

The authors declare that the research was conducted in the absence of any commercial or financial relationships that could be construed as a potential conflict of interest.

## Footnotes

^1^https://youtu.be/F00Sma2BQ4c
